# First-Principle Insight into Ga-Doped MoS_2_ for Sensing SO_2_, SOF_2_ and SO_2_F_2_

**DOI:** 10.3390/nano11020314

**Published:** 2021-01-26

**Authors:** Wenjun Hou, Hongwan Mi, Ruochen Peng, Shudi Peng, Wen Zeng, Qu Zhou

**Affiliations:** 1 College of Engineering and Technology, Southwest University, Chongqing 400715, China; hwj2401@163.com (W.H.); hongwanmi@163.com (H.M.); ruochenpeng@163.com (R.P.); 2 Chongqing Electric Power Research Institute, State Grid Chongqing Electric Power Company, Chongqing 401123, China; pengshudi@163.com; 3 College of Materials Science and Engineering, Chongqing University, Chongqing 400044, China

**Keywords:** sulfur hexafluoride, Ga-MoS_2_, density functional theory, adsorption properties

## Abstract

First-principle calculations were carried out to simulate the three decomposition gases (SO_2_, SOF_2_, and SO_2_F_2_) of sulfur hexafluoride (SF_6_) on Ga-doped MoS_2_ (Ga-MoS_2_) monolayer. Based on density functional theory (DFT), pure MoS_2_ and multiple gas molecules (SF_6_, SO_2_, SOF_2_, and SO_2_F_2_) were built and optimized to the most stable structure. Four types of Ga-doped positions were considered and it was found that Ga dopant preferred to be adsorbed by the top of Mo atom (T_Mo_). For the best adsorption effect, two ways of SO_2_, SOF_2_, and SO_2_F_2_ to approach the doping model were compared and the most favorable mode was selected. The adsorption parameters of Ga-MoS_2_ and intrinsic MoS_2_ were calculated to analyze adsorption properties of Ga-MoS_2_ towards three gases. These analyses suggested that Ga-MoS_2_ could be a good gas-sensing material for SO_2_ and SO_2_F_2_, while it was not suitable for SOF_2_ sensing due to its weak adsorption. This work provides a theoretical basis for the development of Ga-MoS_2_ materials with the hope that it can be used as a good gas-sensing material for electrical equipment.

## 1. Introduction

SF_6_ is known as a colorless, odorless, non-toxic, and incombustible inert gas [1]. It has an octahedral molecular structure and high chemical stability with short bond length and high bond energy [2]. SF_6_ is used for the insulation of Gas-Insulated-Switchgear (GIS) installations and arc extinguishing dielectric due to its extraordinary arc extinguishing property, insulation performance, and adequate chemical steadiness [3]. During the long running times, GIS installations may get some insulation defects such as metal particle defects, metal protrusion defects, air gap defects, etc. [4]. Insulating defects will initiate partial discharge and lead to the disintegration components of SF_6_, such as SO_2_, SOF_2_, and SO_2_F_2_, etc. [5]. On the one hand, some decomposition products are corrosive, which may cause certain damages to the equipment [6,7]. On the other hand, the stability and insulation of the decomposition gases are far inferior than that of SF_6_ [8]. In order to ensure the stable and safe operation of GIS equipment, the specific types and degree of equipment defects could be evaluated by measuring the types and concentrations of SF_6_ disintegration components [9]. At present, one of the main methods to detect SF_6_ disintegration products is the gas sensor detection method [10]. MoS_2_ monolayer is a new member of two-dimensional materials like graphene, which has unique physical, chemical, and electrical characters [11]. According to previous papers, MoS_2_ monolayers have a bandgap of 2.06 eV, suitable carrier fluidity, and high thermal stability [12,13]. In general, its unique structure makes it exhibit good gas sensitivity and adsorption characteristics [14]. Furthermore, published literature already proved that some metal or non-metal doping on MoS_2_ monolayers could further enhance their sensitivity to gas molecules [15,16]. The reason was the fit doped atoms regulate the electrons on the surface of MoS_2_ monolayers and improve the electron density of the doping points to realize the orbital interaction between the doped atoms and some atoms of the gas molecules [17]. Wang J.X. et al. developed a hydrothermal synthesis of Au-MoS_2_ microspheres, whose conductivities were better than that of the intrinsic MoS_2_ material [18]. Abbas H.G. et al. modified MoS_2_ with non-metallic atoms N and P, and tested the adsorption of O_2_ and NO. They found that the adsorption effects of modified N-MoS_2_ and P-MoS_2_ were stronger than that of intrinsic MoS_2_ [19]. Ga is a common semiconductor dopant, which is used as the dopant of nitrogen, arsenic, phosphorus, and other elements. However, as far as we know, the Ga-MoS_2_ monolayer has not been reported for its application in SF_6_ decomposition products detection.

In this paper, Ga-MoS_2_ was selected as sensitive material for adsorption of three typical SF_6_ disintegration components (SO_2_, SOF_2_, and SO_2_F_2_). We performed density functional theory (DFT) method and simulated the adsorption of three gas molecules onto the intrinsic MoS_2_ and Ga-MoS_2_, aiming to shed light on its adsorption ability [20,21]. The optimal configuration of each structure, adsorption energy, charge conversion, density of states (DOS), and band structure were calculated through the DFT method [22]. Furthermore, the simulation results were compared, and the conclusions of material adsorption performance were obtained.

## 2. Computational Details

Our calculations were designed to qualitatively analyze the effects of Ga doping on sensing properties of MoS_2_ at the atomic level. The calculation method was basically consistent with the previous research, so that the results were comparable. The adsorption between MoS_2_-based materials and gas molecules were studied with the DFT method, which is one of the most effective ways to predict the performance of materials by calculating their electronic structures [23]. The molecular spin-polarized algorithms were achieved using DMol^3^ package of Material Studio (MS). The generalized gradient approximation (GGA), which is the Perdew–Burke–Ernzerhof (PBE) functional, was chosen to figure out the doping and hybridization between electrons [24,25]. Within the function of MS software, the double numerical plus polarization (DNP) was utilized as the atomic orbital base install. Meanwhile, the DFT semi-core pseudopotential (DSSP) method was selected to deal with the influence of core electron relativity [26,27,28].

The Brillouin zone of MoS_2_ monolayer systems was sampled with k-point of 5 × 5 × 1, and Self-Consistent Field (SCF) convergence criterion was set to 1 × 10^−6^ Ha [29]. The convergence criteria of geometry optimizations were set to 5 × 10^−3^ A for displacement, 2 × 10^−3^ Ha/Å for force, and 10^−5^ Ha for energy [30]. A 4 × 4 × 1 MoS_2_ monolayer supercell was built, including 16 Mo and 32 S atoms with a vacuum zone of 15 Å [31].

In this paper, the difficulty of doping could be judged by analyzing the energy of formation, adsorption energy, and charge transfer capacity.

We obtained the energy of formation ($E_{form}$) through the calculation:

(1) $$E_{form}=E_{Ga-MoS_{2}}-E_{MoS_{2}}-E_{Ga}$$

$E_{Ga-MoS_{2}}$ is the energy of the system after Ga doping. $E_{MoS_{2}}$ and $E_{Ga}$ denote the energy of pure MoS_2_ and Ga atom, respectively [32,33].

The adsorption energy ($E_{ad}$) of each gas molecules on Ga-MoS_2_ monolayer were calculated by the following equation:

(2) $$E_{ad}=E_{Ga-MoS_{2}/gas}-E_{Ga-MoS_{2}}-E_{gas}$$

$E_{Ga-MoS_{2}/gas}$ is the total energy of gas-adsorbed Ga-MoS_2_ monolayers and $E_{gas}$ represents the energy of gas molecules before adsorption [34,35].

Mulliken charge characterized the number of electrons carried by gas molecules adsorbed. It was utilized to calculate the charge transfer between Ga-MoS_2_ and gas molecules. The definition of charge transfer ($Q_{t}$) used in this paper is:

(3) $$Q_{t}=Q_{adsorbed\left(gas\right)}-Q_{isolated\left(gas\right)}$$

$Q_{adsorbed\left(gas\right)}$ and $Q_{isolated\left(gas\right)}$ mean the number of charges carried by gas molecules before and after adsorption. Normally, the figure of $Q_{adsorbed\left(gas\right)}$ is zero. If electrons are shifted from gas molecules to Ga-MoS_2_ monolayers, $Q_{t}$ is positive [36,37,38].

## 3. Results and Discussion

### 3.1. Geometric Structure of Gas Molecules, MoS_2_, and Ga-MoS_2_ Monolayer

**Figure 1:**
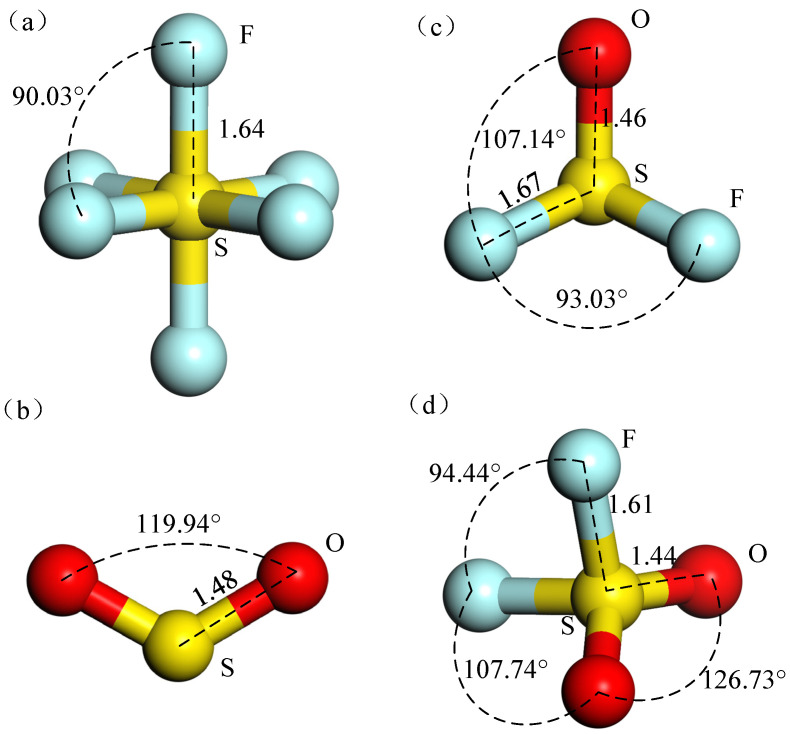
Structures of (**a**) SF_6_, (**b**) SO_2_, (**c**) SOF_2_, (**d**) SO_2_F_2_.

First, the geometric structures of SF_6_, SO_2_, SOF_2_, and SO_2_F_2_ were optimized to their steadiest configuration before studying their adsorption. Figure 1 displays the four structures and the information of bond lengths and angles are shown in Table 1.

**Figure 2:**
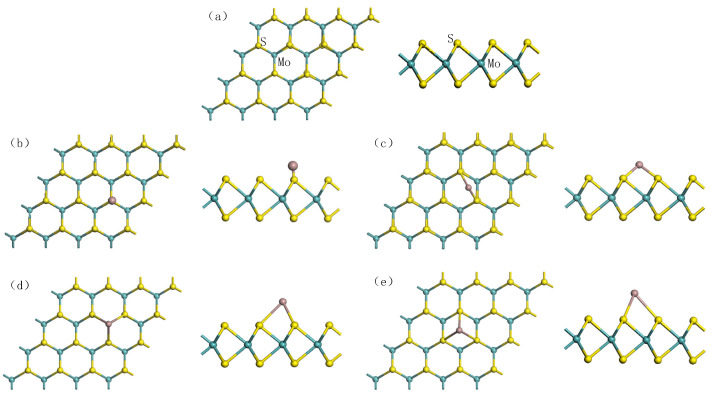
Structure of (**a**) pure MoS_2_, (**b**) T_S_ site, (**c**) B_S-S_ site, (**d**) T_Mo_ site, (**e**) T_H_ site.

**Figure 3:**
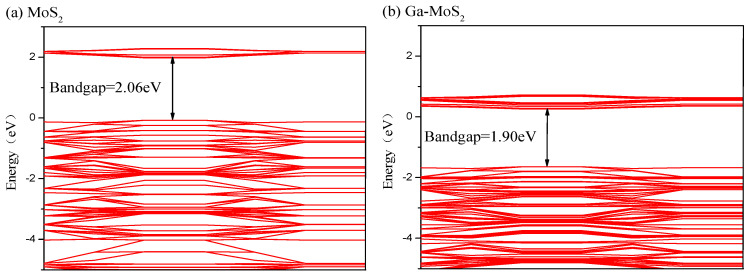
The energy band of (**a**) MoS_2_ (**b**) Ga-MoS_2_ system.

A SF_6_ molecule has a regular octahedral structure with six F atoms arranged symmetrically around an S atom. It is difficult to accumulate enough energy for collision ionization because of the large molecular diameter of SF_6_. When electrons attach and collide with SF_6_ molecules, energy loss will increase and further weaken its ionization ability. Form Figure 1b, the SO_2_ molecule had structure presenting a highly symmetrical “V” shape. SOF_2_ molecule presented a tetrahedral structure, as shown in Figure 1c. SO_2_F_2_ molecule had a similar structure to SOF_2_. Figure 1d indicates that SO_2_F_2_ was symmetrical about the plane where the mid-perpendicular line of two F atoms or two O atoms were connected. The bond length and bond angle of the optimized structure of these gases were consistent with the previous reference [39]. The optimized pure MoS2 monolayer is shown in Figure 2a. The bandgap of the optimized structure is displayed in Figure 3a. The energy gap was 2.06 eV, which was less than the experimental value (3.2 eV) and consistent with the simulation value (2.06 eV) [40]. It indicates that the energy required for the electrons to jump between the valence band and conduction band was large and the electrons were difficult to be excited.

Four types of Ga-doped positions on pure MoS_2_ monolayers were considered, which were T_S_ (at the top of the S atom) in Figure 2b, B_S-S_ (the bridge site between two S atoms) in Figure 2c, T_Mo_ (at the top of the Mo atom) in Figure 2d, and T_H_ (above the hexagonal ring center of MoS_2_) in Figure 2e, respectively [41].

As shown in Table 2, we calculated $E_{form}$ for the four Ga doping models. It was evident that the most favorable doping position had the lowest formation energy, which means that the reaction occurred most easily [42,43]. The T_Mo_ site had the smallest formation energy of −1.75 eV. Therefore, for the optimization model obtained by the four ways Ga can be doped on MoS_2_, the optimization model of T_Mo_ position was the best. From the bond-forming parameters, Ga atoms preferred to approach MoS_2_ monolayers from the top of Mo atoms. Three of Ga-S bonds were formed between Ga and MoS_2_ monolayers, all of which were closed to 2.70 Å in length, indicating the strong interaction between Ga and S atoms. The results show that the surface structure of MoS_2_ did not change much with the doping of Ga. The bond angles of Mo-S-Mo and S-Mo-S were 81.225° and 82.445°, respectively, which had little change from 81.962° before doping. Simultaneously, the distance between the Ga atom and Mo atom was a bit long (3.55 Å), showing the inferior attractive force between them. In conclusion, Figure 2d displays the most stable structure, which is the optimal model used for subsequent adsorption calculations.

**Figure 4:**
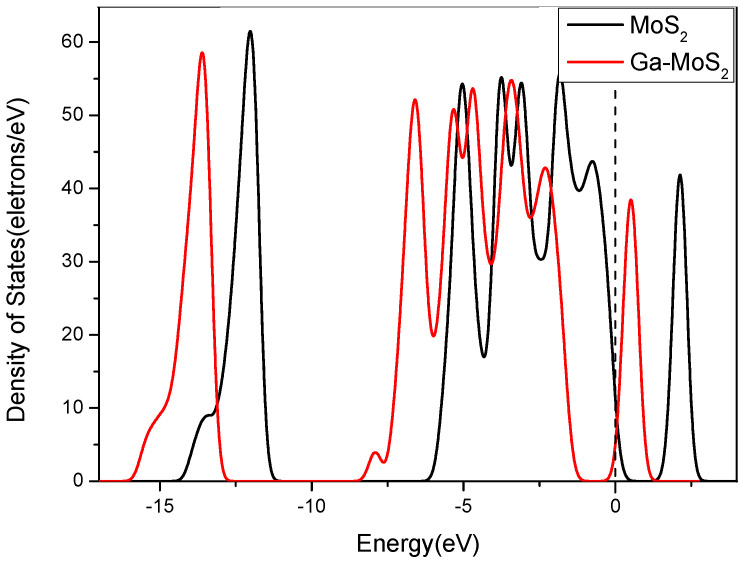
The density of states (DOS) curves comparison before and after doping. The dotted lines are Fermi level.

Figure 3 displays the bandgap structures comparison before and after the Ga doping of MoS_2_. Besides, to compare more intuitively the similarities or differences of electronic structures before and after Ga doping, the DOS contrast was carried out as displayed in Figure 4. In Figure 3, the bandgap between the valence band and conduction band decreased to 1.90 eV. The separation distance corresponds to the width between the two peaks near the Fermi level of DOS. Figure 4 shows that the total density of states (TDOS) shifted to the lower energy direction compared with that before Ga doping. From the Fermi level, it can be seen that the distance between the valence band and the conduction band shortened. It shows that the conductivity of Ga-MoS_2_ ameliorated due to the doping of Ga.

### 3.2. Adsorption Analysis of Ga-MoS_2_ Monolayer to Gas Molecules

#### 3.2.1. Adsorption Analysis of MoS_2_ Monolayer to Gas Molecules

**Figure 5:**
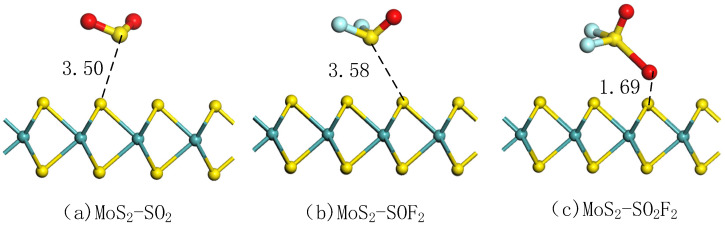
Adsorption structures of MoS_2_ for (**a**) SO_2_ (**b**) SOF_2_ (**c**) SO_2_F_2_.

As Figure 5 shows, the optimized pure MoS_2_ model was used to adsorb the three target gases. The adsorption parameters of three gas adsorption systems listed in Table 3, including adsorption distance (D), $E_{ad}$, and $Q_{t}$ [44]. The adsorption distance between gas molecules and MoS_2_ was large in the adsorption model, which indicates that intrinsic MoS_2_ had weak adsorption capacity for the three gases. It also can see the adsorption of SO_2_, SOF_2_ by MoS_2_ tended to be close to S atom in SO_2_ and SOF_2_ molecules, while SO_2_F_2_ molecule was close to O atom. The S-F bond was slightly elongated in SO_2_F_2_ molecule. As seen in Table 3, the adsorption energies of the three target gases were all positive, indicating that the reaction was endothermic and could not be spontaneous. It proves that the intrinsic MoS_2_ had low adsorption effect on the target gases. The bond angles of SO_2_, SOF_2_, and SO_2_F_2_ were reduced to a certain extent after being adsorbed. The charge transfer values of SO_2_, SOF_2_, and SO_2_F_2_ were negative, and mean that electrons shifted from MoS_2_ surface to gas molecules during the adsorption process. However, the electrons were not active during the adsorption process due to the micro charge transfer in the three adsorption systems. In summary, the intrinsic MoS_2_ had a weak adsorption capacity for the target gases. The improved adsorption performance of MoS_2_ required doping of metal or non-metal atoms.

**Figure 6:**
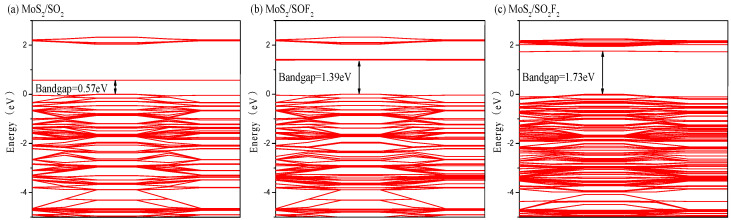
The energy band of (**a**) MoS_2_/SO_2_ (**b**) MoS_2_/SOF_2_ (**c**) MoS_2_/SO_2_F_2_ adsorption system.

The band structures of the adsorption systems corresponding to the three gases were analyzed, as shown in Figure 6. The bandgap of the intrinsic MoS_2_ was 2.06 eV according to the previous calculation. It can be seen from Figure 6a, the bandgap decreased to 0.57 eV after adsorption of SO_2_ molecule, indicating that electrons more easily transited from the valence band to the conduction band, and the conductivity of system significantly improved after adsorption [45]. Figure 6b displays that the bandgap of the SOF_2_ molecule adsorption system reduced by 0.67 eV, illustrating that the conductivity of the system was raised. Figure 6c demonstrates that the bandgap of the SO_2_F_2_ molecule adsorption system diminished by 0.33 eV, the bandgap changed less than the other two gases, and the system’s conductivity did not change evidently. Overall, the bandgap of MoS_2_ decreased most obviously after SO_2_ molecule adsorption.

#### 3.2.2. Selection of Adsorption Modes for Gas Molecules in Ga-MoS_2_ Monolayer

**Figure 7:**
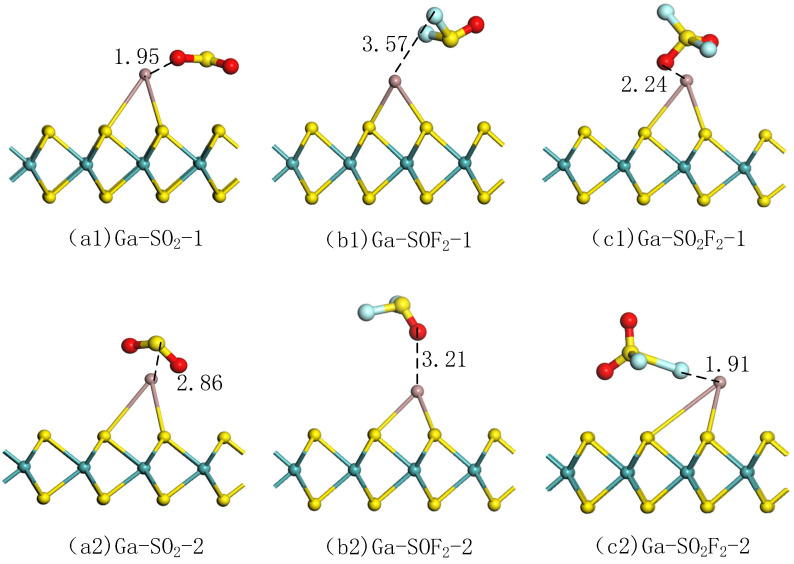
Structure of (**a1**,**a2**) SO_2_ adsorption mode, (**b1**,**b2**) SOF_2_ adsorption mode, (**c1**,**c2**) SO_2_F_2_ adsorption mode.

For the best adsorption effect, two ways for SO_2_, SOF_2_, and SO_2_F_2_ to approach the doping model were compared [46], as shown in Figure 7. The parameters of gas adsorption on Ga-MoS_2_ in different approaches are listed in Table 4.

As Figure 7(a1,a2) shows, two types of SO_2_ adsorption models were built: O atom or S atom near the Ga atom. It can be seen that in the adsorption system obtained after optimization by mode 2, approach mode had changed, while the approach mode 1 had not changed, basically. In the two modes, SO_2_ lost electrons and the Ga-MoS_2_ doping model gained electrons. After calculation, the numerical value of the $E_{ad}$ of the adsorption model constructed in mode 2 was more significant than that constructed in mode 1. At the same time, the absolute value of $Q_{t}$ of the former was larger than that of the latter, and both are negative. It shows that the adsorption process approached in mode 2 was more intense. It also displays that Ga-MoS_2_ was more inclined to mode 2 for the adsorption of SO_2_. Therefore, the adsorption structure obtained by mode 2 was selected as the best structure to facilitate follow-up analysis.

It could be obtained from the above analysis that the adsorption process in the way that S atoms approach the doping model was difficult to occur. This paper adopts two gas approach modes for the adsorption of SOF_2_. The one was mode 1 that approaches F atoms’ doped structure. The other was mode 2, which was approaching the doped structure with O atoms as Figure 7(b1,b2) shows. After optimization calculations, the two adsorption modes were basically unchanged, but the adsorption distance was relatively long. The doping model had a weaker adsorption effect on SOF_2_ for both the approach methods. Both of their adsorption energies were positive, and the amount of charge transfer was very small and close to zero. It proved the adsorption effect of Ga-MoS_2_ on SOF_2_ was not ideal. However, considering the completeness of the analysis and comparison, mode 2, which has lower adsorption energy, was selected in this paper for the subsequent calculation.

Similarly, for the adsorption of SO_2_F_2_, two adsorption methods were selected in this paper. One was to use O atoms to approach the doped structure in Figure 7(c1) and the other was to use F atoms to approach the doped model in Figure 7(c2). It can be seen that the two adsorption methods had not changed to a large extent after optimization calculation. In addition, both SO_2_F_2_ and Ga-MoS_2_ had a certain degree of deformation in mode 2. Among them, an S-F bond of the SO_2_F_2_ was elongated, and the bond between Ga and MoS_2_ was also elongated, which shows that the adsorption process was relatively strong. In comparison, mode 2 had larger adsorption energy and charge transfer amount than mode 1, which also proved that the adsorption process was stronger. In summary, the adsorption model obtained by mode 2 was better for Ga-MoS_2_ adsorption of SO_2_F_2_, so it was selected as the subsequent energy band and DOS analysis.

In order to show the varieties of structural parameters before and after adsorption, D, $E_{ad}$, $Q_{t}$ and some structural parameters of mode 2 are listed in Table 5. Among all the adsorption models of Ga-MoS_2_, the $E_{ad}$ of SO_2_ and SO_2_F_2_ were both negative, indicating that Ga-MoS_2_ could adsorb the above two gases more stably. At the same time, the $E_{ad}$ of SOF_2_ was positive, which suggests that Ga-MoS_2_ had some difficulty in adsorption of SOF_2_.

#### 3.2.3. Energy Band and DOS Analysis

**Figure 8:**
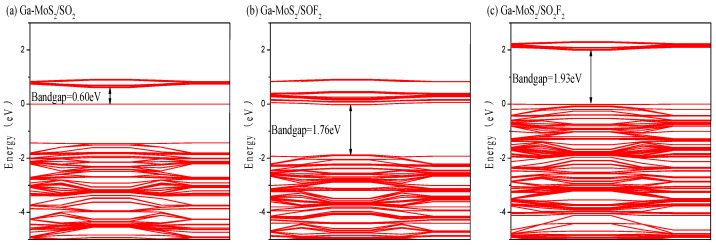
The energy band of (**a**) Ga-MoS_2_/SO_2_ (**b**) Ga-MoS_2_/SOF_2_ (**c**) Ga-MoS_2_/SO_2_F_2_adsorption system.

For the sake of further discussions about electronic characteristics of the model obtained by adsorption, the energy band structure and DOS of the three adsorption models were analyzed [47,48]. Figure 8 shows the band structure diagram of the adsorption system obtained by pure MoS_2_ and Ga-MoS_2_ adsorbing SO_2_, SOF_2_, and SO_2_F_2_.

It can be seen from the above description that the bandgap of Ga-MoS_2_ without adsorbed gas was 1.90 eV. Figure 8a displays the energy band structure diagram of the adsorption system after Ga-MoS_2_ adsorbed SO_2_. The energy bandgap of the system reduced to 0.60 eV, which shows that the adsorption of SO_2_ could make the electrical conductivity of the material promoted to a certain extent. From Figure 8b, it can be seen that after SOF_2_ adsorption, the bandgap of the adsorbed system dropped to 1.76 eV. This indicates that electrons could more likely transition from the valence band to the conduction band than the unabsorbed system. As a result, the conductivity of the material had been improved. Figure 8c displays that after the doped structure adsorbs SO_2_F_2_, the bandgap of the adsorption system becomes 1.93 eV, which is an increase of 0.03 eV compared to the bandgap of the system without gas adsorption. The energy minimum band distance increases slightly. The conductivity of the entire system decreases, but the decrease in the conductivity of the system was very limited due to the small bandgap increase. Thus, the adsorption of SO_2_F_2_ hardly influences the conductivity of Ga-MoS_2_ monolayer.

To sum up, the conductivity of the material could be improved to varying degrees by adsorbing SO_2_ and SOF_2_. At the same time, adsorption of SO_2_F_2_ reduced slightly the conductivity of the material.

**Figure 9:**
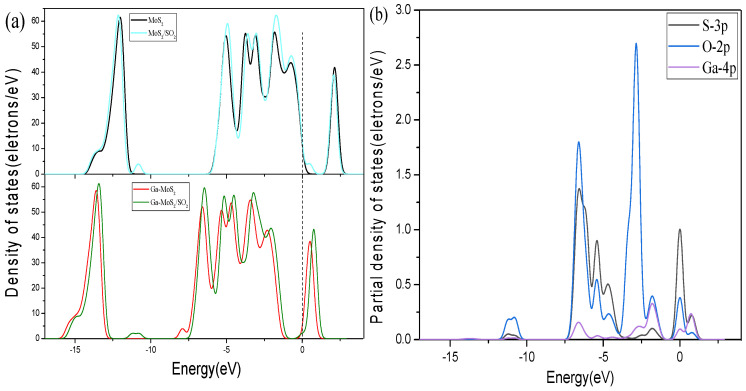
Comparative analysis of (**a**) DOS and (**b**) PDOS for SO_2_ system. The dotted lines are Fermi level.

**Figure 10:**
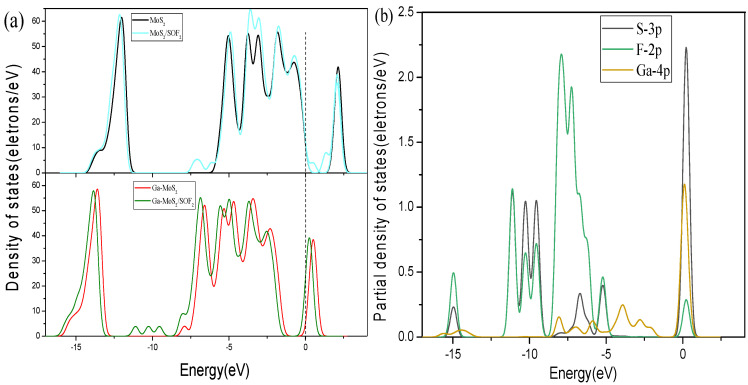
Comparative analysis of (**a**) DOS and (**b**) PDOS for SOF_2_ system. The dotted lines are Fermi level.

**Figure 11:**
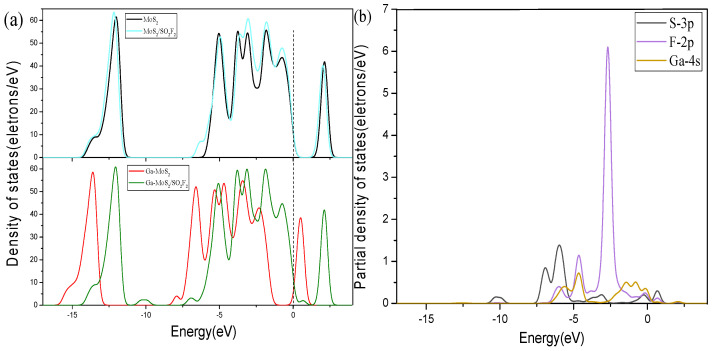
Comparative analysis of (**a**) DOS and (**b**) PDOS for SO_2_F_2_ system. The dotted lines are Fermi level.

Figure 9, Figure 10 and Figure 11 show the DOS of Ga-MoS_2_ for SO_2_, SOF_2_, and SO_2_F_2_ adsorption structures, divided into TDOS and partial density of states (PDOS).

Figure 9a represents the comparison of the TDOS of the system before and after the adsorption of SO_2_. As shown, the TDOS of Ga-MoS_2_ had eight peaks before adsorption of SO_2_. After the adsorption of SO_2_, the adsorption system added a new peak near −10 eV, which indicates that the electron orbit of the adsorption system had changed during the adsorption process. At the same time, the TDOS of the system after adsorption was slightly shifted to the right compared with that before the adsorption. After the adsorption of SO_2_, the peak value of TDOS near 1 eV had increased, and electrons gathered at the bottom of the conduction band. So, the conductivity of the material had been improved to a certain extent. According to the PDOS of Ga-MoS_2_ for SO_2_ adsorption displayed in Figure 9b, the DOS of the listed atoms were mainly distributed in the range of −7.5~0 eV. The hybridization primarily occurred between the 4p orbital of Ga and the 2p orbital of O, and the PDOS of −7.5~−5 eV and −2.5~2.5 eV regions with varying degrees of overlap. At the same time, the 3p orbital of S had the most DOS distribution at the Fermi level. It can be inferred that S had the greatest influence on the electrical conductivity of the entire material in this adsorption system.

Figure 10a displays the TDOS changes of the system before and after SOF_2_ adsorption. The TDOS of the Ga-MoS_2_ system after adsorption had three new peaks. Additionally, the TDOS after adsorption was slightly moved to the left compared to the system before adsorption. The TDOS at the Fermi level had the obvious increase, meaning that the electrical conductivity of the entire system had been improved after being gas adsorbed. The electrons were more likely to jump to the conduction band. The PDOS of the system could be obtained in Figure 10b. The S-3p, F-2p, and Ga-4p orbitals mainly distributed in the range of −12.5~−5 eV. The hybridization of the system was mostly between the 4p orbital of Ga and the 2p orbital of F. At the same time, it can be seen that in this adsorption system, the 3p orbital of S has the most DOS distribution at the Fermi level. It be concluded that, in this system, S had the strongest influence on the electrical conductivity of the material.

From Figure 11a, the TDOS of the entire system had a more obvious shift to the right after SO_2_F_2_ was adsorbed. Focusing on the Fermi level, it is found that the DOS at the Fermi level does not change much before and after adsorption, which indicates that the conductivity of the entire system changed slightly after adsorption of SO_2_F_2_. Figure 11b shows the PDOS of the adsorption system. The DOS of each atom was mainly distributed in the range of −7.5~0 eV. The 4s orbital of Ga and the 2p orbital of F overlap obviously, suggesting that the hybridization of the above-mentioned two orbitals was mainly during the adsorption process. At the same time, it can be seen that in this adsorption system, at the Fermi level, the 4s orbital state density distribution of Ga is the largest, which means that in this adsorption system, the electrical conductivity of the material is mainly affected by Ga atom.

## 4. Conclusions

In this work, theoretical calculations were carried out to study the adsorption properties of Ga-MoS_2_ for SO_2_, SOF_2_, and SO_2_F_2_. The positions of Ga doping were considered. The two ways of gas molecules to approach the doping model were compared. The adsorption parameters, energy bands, and DOS were calculated and analyzed. The main conclusions are as follows:

1. Ga dopant was most likely to be adsorbed onto the MoS_2_ monolayer through T_Mo_ site.
2. The intrinsic MoS_2_ had a weak adsorption capacity for the target gases.
3. SO_2_ molecule tends to approach the doped model with S atoms. SOF_2_ molecule prefers to approach the doped model with O atoms. SO_2_F_2_ molecule is likely to approach the doped model with F atoms.
4. The conductivity of the material could be improved to varying degrees by adsorbing SO_2_, SOF_2_, while adsorption of SO_2_F_2_ had little effect on the conductivity of the material. The $E_{ad}$ of SO_2_ and SO_2_F_2_ were both negative, indicating that Ga-MoS_2_ could adsorb the above two gases more stably. The $E_{ad}$ of SOF_2_ is positive, which proves that Ga-MoS_2_ had some difficulty in adsorption of SOF_2_. The Ga-MoS_2_ can be used as an excellent gas-sensing material for SO_2_ and SO_2_F_2_ molecules.

## Figures and Tables

**Table 1:** Structural parameters of gas molecules.

Gas Molecule	Bond Length (Å)		Bond Angle (°)	
SF_6_	S-F	1.64	F-S-F	90.3
SO_2_	S-O	1.48	O-S-O	119.94
SOF_2_	S-F	1.67	O-S-F	107.14
	S-O	1.46	F-S-F	93.039
SO2F_2_	S-F	1.61	O-S-O	126.73
	S-O	1.44	O-S-F	107.74
	-		F-S-F	94.44

**Table 2:** Formation energy of the four Ga-doping models.

Doping Sites	T_S_	B_S-S_	T_Mo_	T_H_
$E_{form}$(eV)	−1.70	−1.74	−1.75	−1.60

**Table 3:** Parameters of adsorption of the target gases on MoS_2_ surface.

Gas Molecules	D (Å)	$E_{ad}(\mathrm{eV})$	$Q_{t}(e)$	Structure	
SO_2_	3.50	22.34	−0.04	∠ O-S-O	112.64
SOF_2_	3.58	22.41	−0.04	∠ O-S-F ∠ F-S-F	104.10 95.73
SO_2_F_2_	1.69	25.03	−0.68	∠ O-S-O ∠ O-S-F ∠ F-S-F	100.17 95.38 94.08

**Table 4:** Parameters of gas adsorption on Ga-MoS2 in different approaches.

Parameters	SO2		SOF2		SO2F2	
Approach	Mode 1	Mode 2	Mode 1	Mode 2	Mode 1	Mode 2
D (Å)	Ga-O:1.95	Ga-S:2.86	Ga-F:3.57	Ga-O:3.21	Ga-O:2.24	Ga-F:1.91
$E_{ad}$(eV)	−0.61	−0.67	0.36	0.27	−0.15	−0.63
$Q_{t}$(e)	−0.40	−0.42	−0.01	0.01	−0.59	−0.60

**Table 5:** The parameters for the target gases on the Ga-MoS_2_ surface.

Gas Molecule	D (Å)	$E_{ad}(\mathrm{eV})$	$Q_{t}(e)$	Gas Structure		d_Ga-S_ (Å)	∠ S-Ga-S (°)
SO_2_	2.30	−0.67	−0.42	∠ O-S-O	105.53	3.32	52.80
SOF_2_	3.21	0.27	0.01	∠ O-S-F	106.76	2.66	71.19
				∠ F-S-F	93.543		
SO_2_F_2_	1.91	−0.63	−0.60	∠ O-S-O	121.60	2.94	48.62
				∠ O-S-F	105.25		
				∠ F-S-F	88.32		

## Data Availability

The data is available on the request from corresponding author.
